# Supplementary material for: Complex‐centric proteome profiling by SEC‐SWATH‐MS
Source: Mol Syst Biol. 2019 Jan 14;15(1):e8438. doi: 10.15252/msb.20188438 (PMC6346213; doi:10.15252/msb.20188438)
Supplement: Supplementary file 8 — Dataset EV7 [file MSB-15-e8438-s008.zip › feature_plots_string/Q9NX09.pdf]

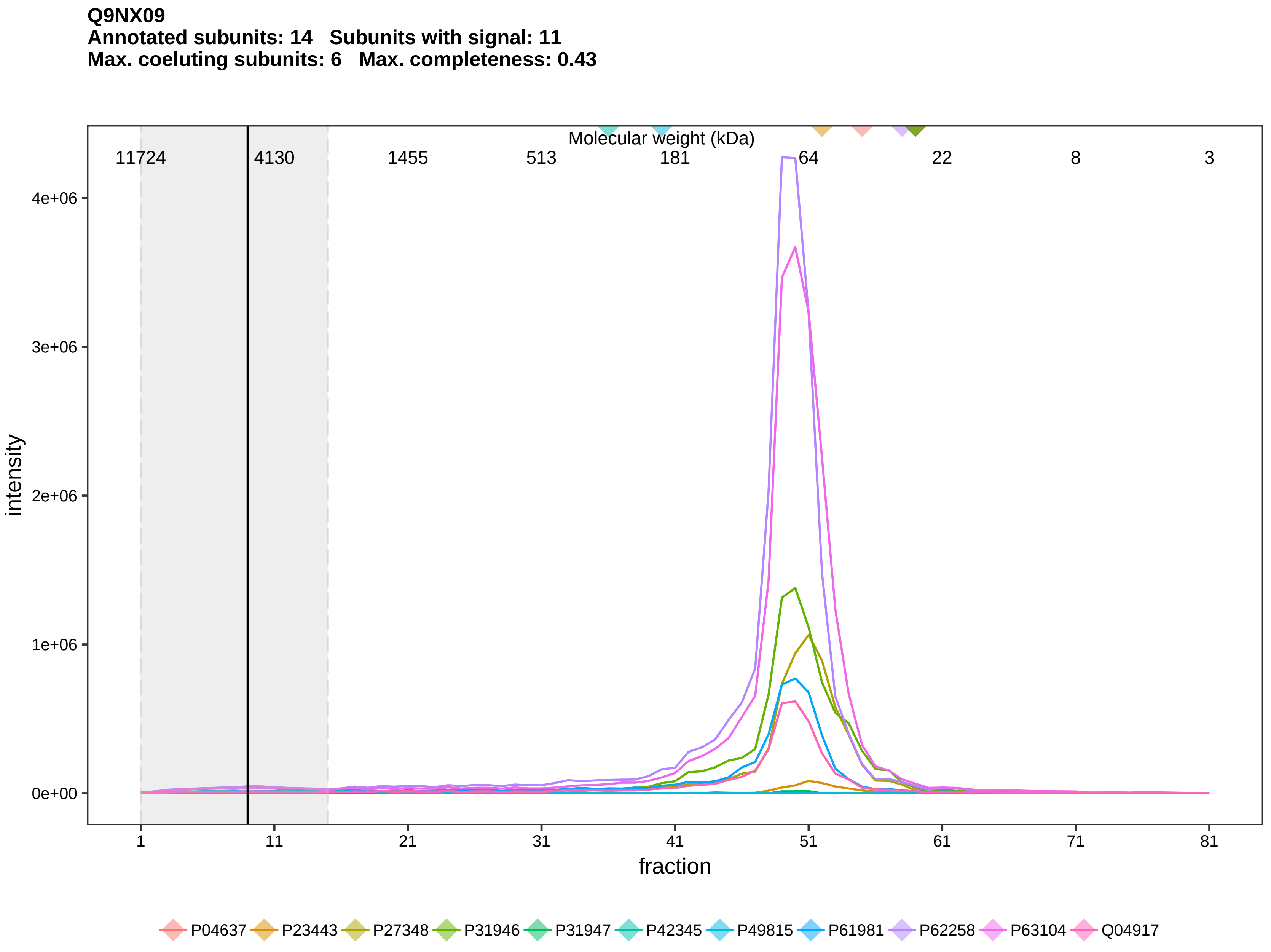
Q9NX09
Annotated subunits: 14   Subunits with signal: 11
Max. coeluting subunits: 6   Max. completeness: 0.43
Molecular weight (kDa)
11724
4130
1455
513
181
64
22
8
3
4e+06
3e+06
2e+06
1e+06
0e+00
intensity
1
11
21
31
41
51
61
71
81
fraction
P04637
P23443
P27348
P31946
P31947
P42345
P49815
P61981
P62258
P63104
Q04917
